# Influence of the Coordinated Ligand on the Optical and Electrical Properties in Titanium Phthalocyanine-Based Active Films for Photovoltaics

**DOI:** 10.3390/ma16020551

**Published:** 2023-01-06

**Authors:** María Elena Sánchez Vergara, Luisa Fernanda Villanueva Heredia, Leon Hamui

**Affiliations:** Facultad de Ingeniería, Universidad Anáhuac México, Avenida Universidad Anáhuac 46, Col. Lomas Anáhuac, Huixquilucan 52786, Estado de Mexico, Mexico

**Keywords:** organic semiconductor, titanium phthalocyanine, active film, optical properties, electrical properties

## Abstract

Tetravalent titanyl phthalocyanine (TiOPc) and titanium phthalocyanine dichloride (TiCl_2_Pc) films were deposited via the high-vacuum thermal evaporation technique and subsequently structurally and morphologically characterized, to be later evaluated in terms of their optoelectronic behavior. The IR and UV-vis spectroscopy of the films displayed α- and β-phase signals in TiOPc and TiCl_2_Pc. Additionally, the UV-vis spectra displayed the B and Q bands in the near-UV region of 270–390 nm and in the visible region between 600 and 880 nm, respectively. The films presented the onset gap (~1.30 eV) and the optical gap (~2.85 eV). Photoluminescence emission bands at 400–600 nm and 800–950 nm are present for the films. One-layer ITO/TiCl_2_Pc or TiOPc/Ag and two-layer ITO/PEDOT:PSS/TiCl_2_Pc or TiOPc/Ag planar heterojunction devices with poly(3,4-ethylenedioxythiophene) polystyrene sulfonate (PEDOT:PSS) deposited by the spin-coating technique were constructed. In these devices, an electrical activation energy between 0.18 and 0.21 eV and a refractive index between 1.14 and 1.44 were obtained. The devices presented a change in the J–V curves for the illuminated and darkness conditions, as much as 1.5 × 10^2^ A/cm^2^, related to the device architecture and phthalocyanine ligand. The latter indicates that the films should be used for optoelectronic applications.

## 1. Introduction

Nowadays, the use of organic semiconductors with charge-carrying capabilities for the manufacture of electronic devices is evident [1,2,3]. Organic semiconductors show promise for photoconversion through their synthetic variability and their low-temperature processing [2,4]. They have been used in photoelectronic devices and solar cells due to their thermal stability, low cost, and an affordable simple synthesis. The right choice of the base organic structure is a determinant aspect in the design of new organic semiconductors. The conjugated compounds with an aromatic system extension tend to show a bigger interaction with neighboring molecules, thus, favoring the charge transport along the semiconductor layer. On the other hand, the material chemical structure determines the electronic device stability and durability. The p-type semiconductors show low HOMO orbital energy values and present good stability in the air [5,6]. Among organic p-type semiconductors, phthalocyanines (Pcs) are representative macrocycle systems with 42 π aromatic electrons, with a good thermal and chemical stability [1,2,3,4]. MPc molecules are particularly appealing because of their unique optical and electrical properties [7,8,9], allowing for their use in organic optoelectronic device applications and particularly in photovoltaic devices, due to the growing interest in solar energy conversion. The Pcs also acquire unique properties as a consequence of the metallic atom presence within the Pc, such as Cu and Zn [10,11,12,13,14].

**Figure 1:**
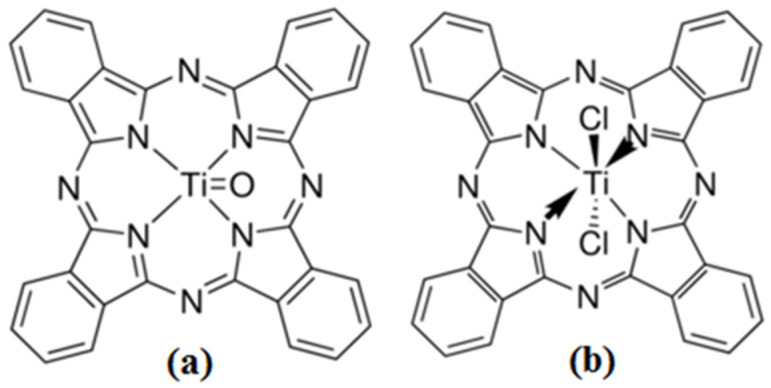
(**a**) Titanyl phthalocyanine (TiOPc) and (**b**) titanium (IV) phthalocyanine dichloride (TiCl_2_Pc).

Along with the already-mentioned Pcs with metallic atoms, there are also phthalocyanines with titanium tetravalent configuration, such as titanyl phthalocyanine (TiOPc) and titanium phthalocyanine dichloride (TiCl_2_Pc) [15,16,17]. They show a p-type transport characteristic, which makes them act as semiconductors. Apparently, a relationship between the charge carrier transport type and their axial ligand also exists. The TiCl_2_Pc has a p-type transport characteristic, where both the axial ligands and the electronegativity of the metal influence the LUMO energy and the charge distribution [15]. The TiCl_2_Pc exhibits emission efficiencies enough to be considered as potential infrared emitters [15,17]. On the other hand, the TiOPc also presents a p-type transport characteristic and it is known to be one of the organic materials that exhibits the largest photo-carrier generation efficiency, which has been successfully tested in laser-printer technologies [13,14,16,17]. Additionally, the TiOPc shows interesting non-linear optical properties with applications for optical disk design [17]. However, related studies for the molecular state of TiOPc and TiCl_2_Pc are still rare and titanium phthalocyanine compounds have not received extensive research and study [18]. This results in a lack of optoelectronic properties’ correlation with their chemical structures, which is much less explored than other Pcs [7,8,9,10,11,12,13,14,15,16,17,18]. Due to that previously mentioned, the objective of this work is to present a comparative study between optical properties and the charge-carrying capability present in each one. The TiOPc and TiCl_2_Pc are non-planar molecules (see Figure 1), which is different to most Pcs (such as CuPc and ZnPc) studied in organic electronics.

This work presents two important differences with respect to other studies carried out on titanium phthalocyanines. The first novelty is the evaluation of the chloride and oxygen ligands’ influence on the optical and electrical properties of TiCl_2_Pc and TiOPc, respectively. Subsequently, the most important novelty is the preparation and the optical and electrical characterization of the film-based planar heterojunction: poly(3,4-ethylenedioxythiophene) polystyrene sulfonate/phthalocyanine (PEDOT:PSS/TiCl_2_Pc or TiOPc), for the determination of the optimal heterostructure to be used as an active layer in photovoltaic devices. The search of organic semiconductors that act as a stable active layer continues to be an interesting theme within the molecular electronics field. This is because of the stability and solubility problems in organic semiconductor films that hinder their application in optoelectronic devices. Moreover, the PEDOT:PSS used for this work is a polymer that, due to its thermal and mechanical stability, has proved its worth in the development of several electronic devices [19]. The aim in this work is to fabricate a highly efficient planar heterojunction by exploiting the benefits of the enhanced electrical conductivity of the PEDOT:PSS with interesting characteristics in TiCl_2_Pc and TiOPc. The resulting electrical characterization of the ITO/PEDOT:PSS/MPc or MPc/Ag devices made it possible to establish their possible applications in electronic and photovoltaic devices.

## 2. Materials and Methods

### 2.1. Materials and Equipment

All reagents and solvents were obtained from commercial suppliers (Merck KGaA, Darmstadt, Germany) and used without further purification. The used compounds were titanyl phthalocyanine (C_32_H_16_N_8_OTi) with a molecular structure shown in Figure 1a and titanium(IV) phthalocyanine dichloride (C_32_H_16_Cl_2_N_8_Ti) with a molecular structure shown in Figure 1b. The IR spectroscopy characterization of powdered materials and films was carried out by means of a Nicolet iS5-FT spectrophotometer (Thermo Fisher Scientific Inc., Waltham, MA, USA), within a 4000–300 cm^−1^ region with an 8 cm^−1^ resolution. Morphological and topographical characteristics were investigated with a ZEISS EVO LS 10 scanning electron microscope (SEM) (Carl Zeiss AG. Jena, Germany) and with a Nano AFM atomic force microscope (Nanosurf AG, Liesta, Switzerland) using an Ntegra platform for the films deposited on the PET substrate. The X-ray diffraction (XRD) analysis was performed with the θ–2θ technique using a Bragg-Brentano geometry with a Siemens D5000 diffractometer (Siemens, Aubery, TX, USA) and working with Cu-Kα (λ = 0.15405 nm) radiation. The samples were measured at 0.4°/min, interval 2–70°. The absorbance and transmittance of the films on glass were obtained in a 200–1100 nm wavelength range, on a UV-Vis 300 Unicam spectrophotometer (Thermo Fisher Scientific Inc., Waltham, MA, USA), respectively. Additionally, a Gaertner L117 Ellipsometer equipped with a He-Ne laser (λ = 632.8 nm) was used to obtain the refractive index, the optical properties, and to verify the thickness obtained from the evaporator quartz microbalance. Photoluminescence (PL) was measured using a He-Cd laser (Kimmon Koha Co., Ltd., Centennial, CO, USA) with an excitation wavelength of 325 nm and integration time of 100 ms. For the electrical characterization of the devices, ITO and silver were used as anode and cathode, respectively. For this evaluation, a programmable voltage source, a sensing station with lighting and temperature controller circuit from Next Robotix (Comercializadora K Mox, S.A. de C.V., Mexico City, Mexico), and an auto-ranging Keithley 4200-SCS-PK1 pico-ammeter (Tektronix Inc., Beaverton, OR, USA) were employed with a four-point probe collinear method. The evaluation of the electrical behavior of the flexible devices was performed both under illuminated and darkness conditions. Further, it was performed by changing the temperature from 25 °C to 245 °C and the illumination light color.

### 2.2. Thin-Film and Device Fabrication

**Figure 2:**
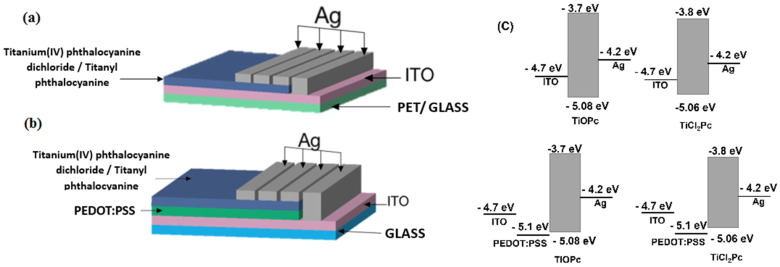
Diagrams of the devices (**a**) ITO/MPc/Ag and (**b**) ITO/PEDOT:PSS/MPc/Ag and (**c**) their energy-level diagrams.

The TiOPc and TiCl_2_Pc were deposited by the high-vacuum thermal evaporation technique onto the different substrates: monocrystalline n-type silicon wafers (c-Si), glass, indium tin oxide (In_2_O_3_·(SnO_2_)_x_)-coated polyethylene terephthalate (PET-ITO) substrate, and ITO-coated glass substrate (glass-ITO). Previously, all substrates, excluding PET-ITO, were cleansed by applying an ultrasonic process, using chloroform, methanol, and acetone, and then dried in vacuum. TiOPc and TiCl_2_Pc were deposited in a high-vacuum evaporation system (Intercovamex, S.A. de C.V., Cuernavaca, Morelos, Mexico) using tantalum crucibles, a vacuum pressure of 1 × 10^−5^ torr, and deposit speed of 4.5 Å/s. Pcs were heated to 300 °C to produce their phase change, which was initially carried out in the gaseous state, so that they would finally be deposited in thin-film form upon contact with the substrates set at room temperature. Due to the different Pcs structure and melting point, the thickness of each film was 138 Å for TiOPc and 31 Å for TiCl_2_Pc. The thickness was monitored using a microbalance quartz crystal monitor, connected to a thickness sensor. For the evaluation of electrical properties, the structures of ITO/MPc/Ag and ITO/MPc/Ag were used in the device setup with PET and glass substrates (see Figure 2a). After depositing the TiOPc and TiCl_2_Pc films, they were subjected to a heat treatment in an oven (Briteg Instrumentos Científicos S.A. de C.V.) for 2.5 h at 300 °C and left to cool for 10 min at room temperature.

To complement the study on the behavior of phthalocyanine films as an active layer, additional devices were fabricated on glass: ITO/PEDOT:PSS/MPc or ITO/MPc/Ag (see Figure 2b). Energy-level diagrams for the fabricated devices are shown in Figure 2c. The poly(3,4-ethylenedioxythiophene)-poly(styrenesulfonate) (PEDOT:PSS) film was deposited by the spin-coating technique as a hole-transporting layer in Smart Coater 200 equipment (Laurell Technologies Corporation, North Wales, PA, USA). The dispersion used for the manufacture of the films consisted of poly(3,4-ethylenedioxythiophene) polystyrene sulfonate (PEDOT:PSS) in 1.1% in H_2_O with neutral pH and high-conductivity grade. The dispersion was deposited on the substrate and the equipment was operated at a constant angular speed of 300 rpm during 10 s and an acceleration of 80 rpm/s, then dried at 80 °C for 3 min. After the deposit of PEDOT:PSS, the TiOPc and TiCl_2_Pc were subsequently deposited by the high-vacuum thermal evaporation technique with the previous deposition parameters and annealed for 2.5 h at 300 °C.

## 3. Results and Discussion

### 3.1. Structural and Morphological Characterization

**Figure 3:**
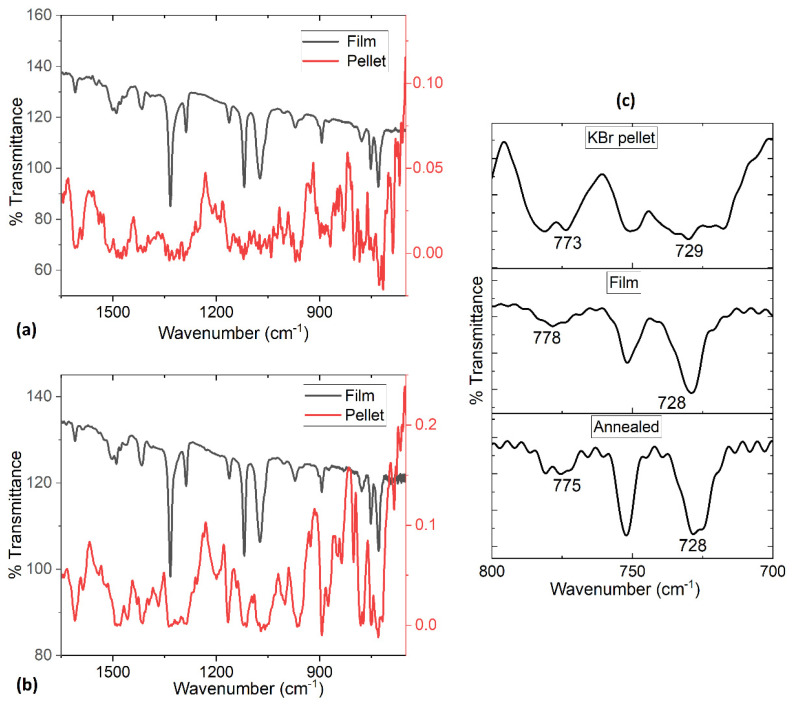
IR spectra of (**a**) TiOPc, (**b**) TiCl_2_Pc in pellet and thin film and (**c**) TiOPc in a range of 700 to 800 cm^−1^.

IR spectroscopy was performed for TiOPc and TiCl_2_Pc, in KBr pellets and in films deposited on a silicon substrate. The above is to establish if any degradation of the material took place during the deposit of the film as a consequence of the Pc sublimation and its subsequent deposition on the substrates. The IR spectroscopy is based on the fact that the Pc bonds have specific vibration frequencies that correspond to the molecule energy levels. In the present study, it is sought that the spectrum of the TiOPc and TiCl_2_Pc in pellets equals the films spectrum, deposited in silicon. In Figure 3a,b and in Table 1, the values found of the representative vibrations of the TiOPc and TiCl_2_Pc structures are shown, both in pellets and in thin film: (i) the band responsible for the pyrrole in-plane stretch vibration in the Pc ring is observed around 1587 and 1335 cm^−1^, (ii) the bands located around 1290, 1166 and 1118 cm^−1^ are the result of the interaction between C of the peripheral rings, with the hydrogen atoms [20], (iii) the band located around 753 cm^−1^ is the interaction in plane of C-H deformation, and (iv) the bands observed around 1610 and 1475 cm^−1^ result from a C=C stretching mode [15,16,17,20]. From the IR spectroscopy analysis, it can be concluded that the signals are present, so there is no thermal degradation. Additionally, the IR spectra were used to identify the different polymorphs in MPcs [7,15,21]. MPcs can exist in various polymorphic forms identified as α, β, γ, δ, ε, and χ phases with the metastable α phase and stable β phase being the most common [7,22,23,24]. The signals are found around 724 cm^−1^ for the α phase and around 777 cm^−1^ for the β phase [15,25,26]. In the case of the TiOPc and TiCl_2_Pc films, the spectrum in KBr displayed the signals of both phases. In the literature, it is mentioned that the phase transition from α to β phase occurs in most metallic phthalocyanine (MPc) films through a temperature exposure from 200 to 300 °C [7,23,27,28,29]. However, as can be seen in Figure 3c, for the non-planar TiOPc, this transformation did not occur, neither when forming the film nor when performing the annealing. Similar results occurred with TiCl_2_Pc in KBr pellet and in film form, also after annealing; the α and β phases of the phthalocyanine were maintained. This result is indicative of the high thermal stability of TiOPc and TiCl_2_Pc. The latter is a high-vacuum evaporation technique that tends to form amorphous films as a consequence of the sublimation and subsequent nucleation and growth process. In the TiOPc and TiCl_2_Pc films, there were practically no changes in orientation and structure.

**Figure 4:**
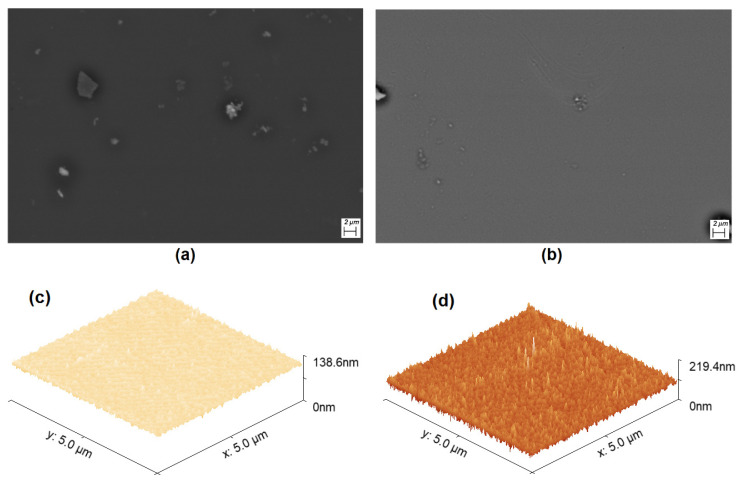
SEM microphotographs of (**a**) TiOPc and (**b**) TiCl_2_Pc films at 5000×. AFM images of (**c**) TiOPc and (**d**) TiCl_2_Pc films.

Another important aspect to consider additionally to the thermal stability is the morphology of the films deposited in terms of their homogeneity, grain size, and impurity level. To verify the above, SEM was performed and, in Figure 4, the microphotographs at 1000x are shown, which allow for the observation of the morphological characteristics of the TiOPc and TiCl_2_Pc films on the glass substrate. With SEM analysis, the particle size can be analyzed, as well as the morphology and the uniformity of the Pc films. It should be noted that the film uniformity is an important factor so that the electric charge transport must remain constant throughout the entire device area. On the contrary, if there are films with heterogeneous morphology, their electric properties decrease because the charge transport is not uniform. In Figure 4a,b, a greater number of particles in the TiOPc film is observed, while the TiCl_2_Pc film is more uniform. In addition, in the film in Figure 4a, larger particles are observed on the surface and even form agglomerates of sizes around 2 μm. The particles at the top of the films are formed as result of the nucleation and growth of the Pcs during deposit. Apparently, the growth of the TiOPc and TiCl_2_Pc films is carried out by the Stranski–Krastanov mode (SK). SK growth describes the formation of complete Pc monolayers, where subsequent 2D growth is unfavorable and 3D island growth continues. Island growth occurs when Pc molecules are more strongly attracted to each other than to the substrate, resulting in 3D growth and Pc films experiencing SK growth [30,31,32]. The higher uniformity in the film with TiCl_2_Pc could generate a greater charge transport, although there are factors that will have to be considered later, such as the topography and roughness of the films. According to the AFM micrographs in Figure 4c,d, the film topography consists of fine and granular particles homogeneously distributed around the film surface. With respect to the roughness, Table 2 shows the results for TiOPc and TiCl_2_Pc films and there is no significant variation between the Root Mean Square (RMS) roughness and the average (Ra) roughness of both films. This is expected considering that the substrate and the film deposit parameters are the same. Additionally, it is important to consider that low roughness is observed for both films, which is considered an advantage for the charge transport and for films interaction in a planar heterojunction for optoelectronic devices (see Figure 2). The TiOPc and TiCl_2_Pc films’ low roughness will make a perfectly defined interface between the films that integrate the devices and will also ease the transport of the charge carriers.

**Figure 5:**
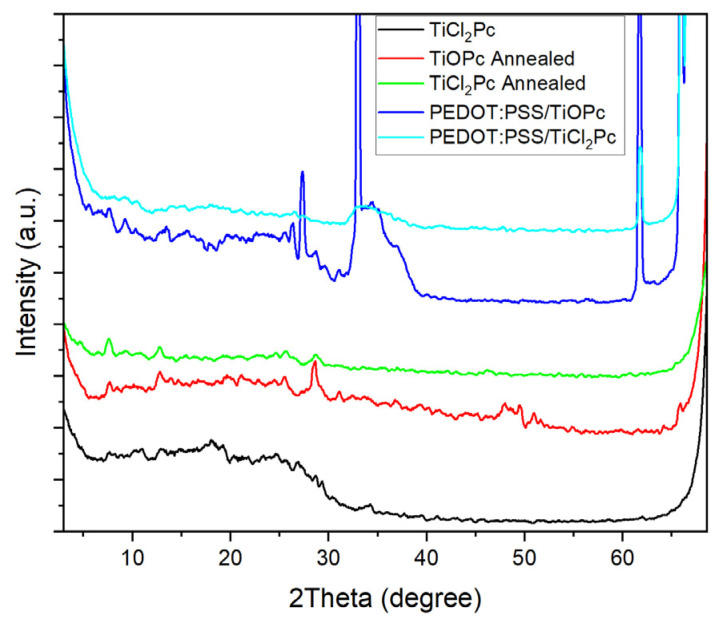
XRD patterns of the TiOPc and TiCl_2_Pc films and devices.

Figure 5 shows the XRD patterns for the TiOPc and TiCl_2_Pc films and devices. In addition, Table 3 shows the XRD peak positions, FWHM, and crystallite size for the TiOPc and TiCl_2_Pc films and devices. By comparing the TiOPc and TiCl_2_Pc annealed films, the contribution of the Pcs and the films can be observed and they are found to be polycrystalline [33,34]. Various peak positions are coincident with a slight shift and with an intensity variation. The most intense peaks can be observed, approximately, at 7.6°, 12.8°, 25.7°, and 28.6°, but for the TiOPc, further peaks are also observed at higher 2θ values. However, by comparing to the literature, there is a shift to higher 2θ values, which may be related to a structural change and arrangement consequence of the thermal annealing. To understand this effect, an XRD pattern for the TiCl_2_Pc film is shown in Figure 5. By comparing to the annealed film, broader peaks and lower 2θ values (Table 3) for the characteristic Pc peaks can be observed and match with the literature [33,34]. XRD patterns for the PEDOT:PSS/TiCl_2_Pc and PEDOT:PSS/TiOPc devices are also shown in Figure 5. First, a very intense peak appearance is observed related to the PEDOT:PSS effect, in particular for the PEDOT:PSS/TiCl_2_Pc, complimentary to that observed for the TiOPc and TiCl_2_Pc annealed films. Further, a shift to higher 2θ values (Table 3) for the characteristic Pc peaks can be observed, indicative of the PEDOT:PSS effect in the Pc deposition. Due to the observed polycrystalline behavior, the film and device crystallite size can be estimated by the Scherrer equation [35,36]:

(1) $$D=\frac{K\lambda }{\beta cos\theta }$$

where *D* is the mean crystallite size of the θ Bragg angle, *λ* the X-ray wavelength, *K* the shape factor (~0.89), and *β* the full width at half maximum (FWHM). Table 3 shows the resulting sizes (~0.2–1 nm), where for the TiCl_2_Pc film, smaller sizes are presented, supporting the observations in Figure 4. On the other hand, the PEDOT:PSS/TiCl_2_Pc and PEDOT:PSS/TiOPc devices had very different results. For the PEDOT:PSS/TiOPc, an interesting increase in crystallite size was observed, but for the PEDOT:PSS/TiCl_2_Pc, an apparent decrease is observed. The latter will affect the optoelectronic properties of the devices compared to the the TiOPc and TiCl_2_Pc annealed films, resulting from the absorption and charge-carrier transport variation.

### 3.2. Evaluation of Optical Properties

**Figure 6:**
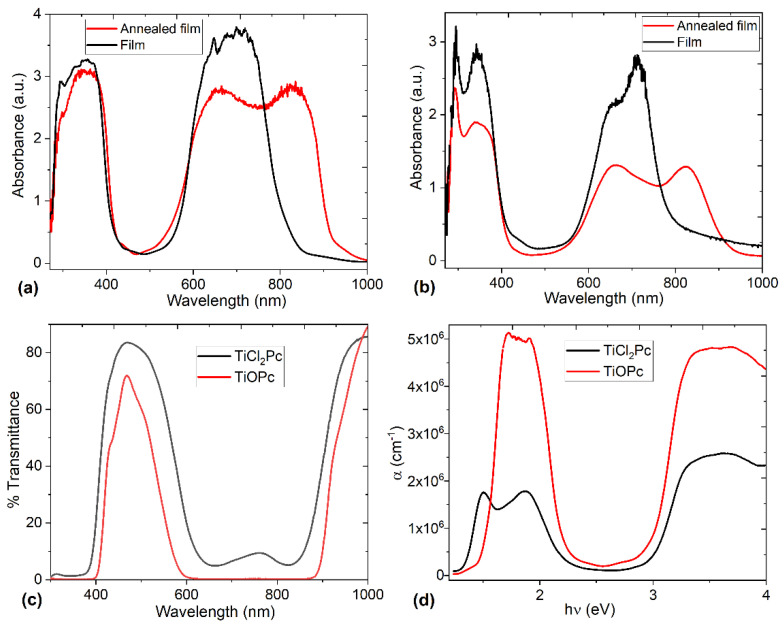
Absorbance spectra of (**a**) TiOPc and (**b**) TiCl_2_Pc films before and after annealing. (**c**) Transmittance and (**d**) absorption coefficient of TiOPc and TiCl_2_Pc films after annealing.

In order to study the optical behavior of TiOPc and TiCl_2_Pc, UV-vis spectroscopy was carried out in the films deposited and annealed. In Figure 6, the UV-vis absorption spectra of TiOPc and TiCl_2_Pc are shown and they are the result of their conjugated π-electron systems and the central titanium overlapping orbitals [7,28,29,37,38,39]. According to the spectra, it is observed that the annealing influences the film’s absorption by its decrease and redshift. The annealing also defines and enhances the electronic transitions, probably due to an arrangement of the Pc molecules forming the films. Additionally, the spectra displayed two strong absorption bands, known as B and Q bands. The B band in the near-UV region of 270–390 nm is assigned to the electronic transition between π and π* (b_2u_ to e_g_) orbitals [7,28,40]. For TiCl_2_Pc, the B band displayed two peaks: (i) the low-energy region (around 340 nm) is due to the π-d transitions between the Pc ring and the titanium atom and (ii) the higher-energy region (around 290 nm) corresponding to d-π* transitions [7,17,41,42]. The Q band in the visible region of the spectrum between 600 and 880 nm represents the π-π* transition (b_1u_ to e_g_) orbitals [7,28,40]. A split of the annealed film’s Q band is observed, probably by the Davydov splitting. The extent of Davydov splitting is related to the degree of available molecules able to participate in electronic transitions, in particular, interactions between the dipole moment transition from adjacent molecules [7,29]. In the annealed film Q band, the high-energy peak (around 660 nm) is related to the electronic transition from π-π* orbitals of the macrocycle, while the low-energy peak (around 820 nm) may be explained as a second π-π* transition, an exciton peak, a vibrational interval, or a surface state [7,28,29]. The position, intensity of these peaks, and the amount of Davydov splitting for the α and β phases in Pcs are different and depend on the molecular orbital overlap. For the two films, the intensity of the higher-energy maximum peak (661 nm) is almost equal to the lower-energy peak (823 nm), with a Davydov splitting amount of 162 nm among the two phases. The above means that for both phthalocyanine films, the α and β phases are present and, apparently, the non-planar TiOPc and TiCl_2_Pc have π-stacked configuration with face-to-face packing [7]. Finally, in the UV-vis spectra, it is observed that for the TiOPc film, greater Q-band absorption and redshift are present. The above is probably due to the oxygen coordination to the titanium atom, compared with the chlorides in the fifth and sixth position of the titanium coordination sphere in the TiCl_2_Pc.

Regarding the transmittance, Figure 6c shows the spectrum for both annealed films. It is observed that for the TiCl_2_Pc film, there is a higher transmittance with respect to the TiOPc film. On the other hand, the spectrum can be split into different regions; the first is the non-absorption region between 380 and 620 nm. The second is the absorption region and it is clearly seen that the minimum in the transmission spectra in a 600–880 nm range is due to the band-to-band transition region, which corresponds to the Q band. Finally, the third region is, again, the non-absorption between 880 and 1000 nm. These results are interesting because the predominant factor in the optical properties of the films is observed to be a consequence of the macrocycle with the titanium atom in the phthalocyanine and not due to its substituent. In addition, the observed wavelength-dependent change in its behavior is an indication of the possible applications as a transparent anode in photodiodes or solar cells, to mention some device types, where electromagnetic radiation is a decisive parameter for its operation.

Reflectance (*R*) and the refractive indices (*n*) in semiconductors films are relevant in the design and analysis of optoelectronic devices [43]. The resulting refractive index from ellipsometry measurements for the studied films was 1.137 and 1.182 for TiOPc and TiCl_2_Pc, respectively. For normal incidence, the reflection coefficient that affects the intensity of the radiation is expressed as [44]:

(2) $$R=\frac{{\left(n-n_{s}\right)}^{2}+k^{2}}{{\left(n+n_{s}\right)}^{2}+k^{2}}$$

where *k* is the attenuation or extinction constant and 𝑛_𝑠_ is the refractive index of the substrate, which, for glass, is 1.52. In the case where *k* = 0, in the transparent range, the reflectance is 0.0208 and 0.0156 for TiOPc and TiCl2Pc, respectively. These films are not perfectly transparent or perfectly reflective and radiation is lost. The losses are manifested through the absorption coefficient (α) given by Equation (3) [18,35]:

(3) $$\alpha =\frac{1}{d}ln\left[\frac{{\left(1-R\right)}^{2}}{2T}+\sqrt{R^{2}+\frac{{\left(1-R\right)}^{4}}{4T^{2}}}\right]$$

where *d* is the film thickness and *T* is the transmittance of the films and, in the case of TiOPc and TiCl_2_Pc thin films with R << 1, the previous expression is expressed as follows [45]:

(4) $$\alpha =\frac{1}{d}ln\left[\frac{1}{T}\right]$$

The spectral behavior of the α for the annealed films in a photon energy range of 1.2–4 eV is depicted in Figure 6d. According to the literature for MPc films [20,21,39,41,46,47], the films have a high α > 10^6^. The traps inside the energy gap can be responsible for the high α, indicating that these films can be used in optoelectronic devices.

To complement the study of the optical behavior in the TiOPc and TiCl_2_Pc films, the energy bandgap was calculated through Tauc’s method used as a standard empirical model [48]. The optical bandgap energy controls the light-absorption efficiency in optoelectronic devices. The calculation to obtain the optical bandgap energy with Tauc’s method is based in the Urbach relation (see Equation (5)), where *h* is Planck’s constant, parameter *B* depends on transition probability, Eg is the bandgap energy, and *n* is dependent on the electronic transition process, where *n = 2* for indirect allowed transitions [46,48,49,50,51].

(5) $$\alpha h\nu =B{\left(h\nu -Eg\right)}^{n}$$

The frequency (*ν*) is experimentally obtained from Equation (5), where *c* is the speed of light and *λ* is the wavelength.

(6) $$\nu =\frac{c}{\lambda }$$

**Figure 7:**
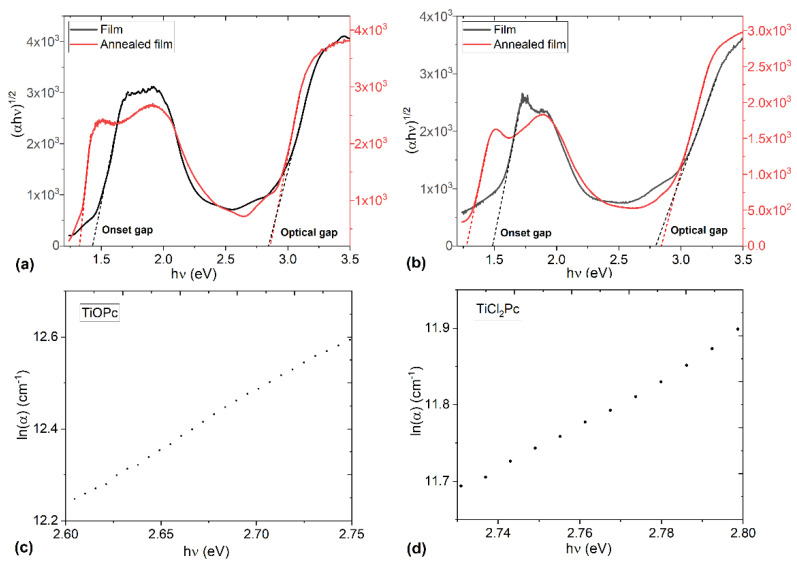
Variation in (αhν)^1/2^ with hν for (**a**) TiOPc and (**b**) TiCl_2_Pc films before and after annealing. Variation in ln(α) with hν for (**c**) TiOPc and (**d**) TiCl_2_Pc films.

The dependence of (αhν)^n^ on *hν* was plotted and the Eg was evaluated from the *x*-axis intercept at (αhν)^1/2^= 0. The Figure 7 plots show two transitions; the first transition is the onset gap $\left(E_{g}^{onset}\right)$ and the second one corresponds to the optical gap $\left(E_{g}^{optical}\right)$ [47] for the films before and after annealing. The results are shown in Figure 7 and Table 4. It is important to observe, in Table 4, that the onset bandgap is slightly less for the TiOPc film and decreases after thermal treatment. However, the optical gap practically does not change after annealing and there is not a change between the two films. In addition, the obtained energy bandgap values are similar to those reported in the literature for TiPcCl_2_ films and other chlorinated phthalocyanine films, such as AlPcCl, GaClPc, and SnPcCl_2_ [18]. Apparently, the ligands and, in general, the titanium atom, not related to the charge transport in the films. The charge transport is mainly related to the molecular packing and the highly aromatic electrons of macrocycle. The electronic transition from π to π* explains that the optical gap and the onset gap are a consequence of several factors, including defects, structure disorder, and traps. According to Alosabi et al. [18], the Urbach energy can be used to determine the defects in the energy gap. The Urbach energy E_U_ can be determined according to Equation (7) [18,36]:

(7) $$\alpha =A_{a}exp\left(\frac{hv}{E_{U}}\right)$$

where, in addition to the parameters defined above, *A_a_* is a constant of the material that conforms to the absorption coefficient at the energy gap. The exponential absorption edge can be interpreted as the exponential distribution of localized states in the energy bandgap [18]. Figure 7c,d displayed the linear relation between ln(*α*) and *hν* for the TiOPc and TiCl_2_Pc films. The values of the Urbach energies were determined from the reciprocal of the slope from this linear relation. The obtained Urbach energy values are 0.40 eV and 0.32 eV for TiOPc and TiCl_2_Pc films, respectively. The Urbach energy value for TiCl_2_Pc is slightly lower than those obtained for MPc films with different metal atoms than the titanium atom (around 0.4 eV) [18].

To evaluate the behavior as an active layer in optoelectronic devices, the TiOPc and TiCl_2_Pc films were deposited on a PEDOT:PSS polymer hole-transporting film. Later, the energy bandgap of these systems was evaluated, before and after annealing, and the results are shown in Table 4. In this case, the annealing process decreased the onset gap and the optical gap, although the values for both systems, PEDOT:PSS/TiOPc and PEDOT:PSS/TiCl_2_Pc, are similar.

**Figure 8:**
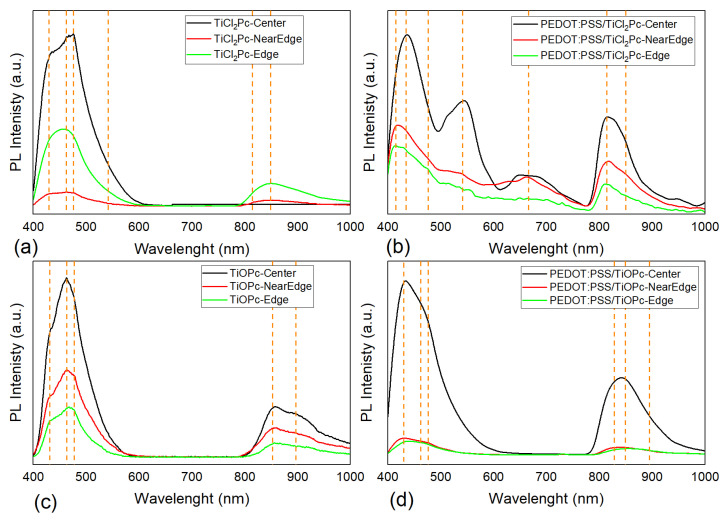
PL spectra of (**a**) TiCl_2_Pc and (**b**) PEDOT:PSS/TiCl_2_Pc and (**c**) TiOPc and (**d**) PEDOT:PSS/TiOPc films.

Additionally, photoluminescence (PL) measurements were conducted on the samples and plotted in Figure 8. The PL emitted by the excitation spot on the films appeared as follows: blue green (TiCl_2_Pc), blue (TiOPc), violet blue (PEDOT:PSS/TiCl_2_Pc), violet blue (PEDOT:PSS/TiOPc), and with an intensity high enough that was observed with the naked eye. As depicted for all the devices, two main broad bands are observed in the 400–600 nm and 800–950 nm ranges. The measurements were conducted for three locations within the sample to evaluate the homogeneity and PL response: at the sample center, near the edge, and the edge. For all spectra, the sample center presents a higher PL intensity. The spectra for all locations are almost similar for most of the samples, maintaining the emission wavelengths due to homogeneity, but the emission intensities vary according to the location along the sample. The latter is mainly related to the location film thickness and arrangement of the molecules, as a consequence of the deposition process. The emission in the blue region (i.e., 430–550 nm) is present in all spectra and related to the phthalocyanine contribution [52,53]. For TiCl_2_Pc (Figure 8a), a maximum emission is observed at 475 nm and for the TiOPc film (Figure 8c) at 460 nm, which is also present for the TiCl_2_Pc film. On the other hand, a shoulder around 430 nm is observed for both films, but more intense for TiCl_2_Pc, mainly related to a singlet exciton recombination. Further, a broadening to 600 nm of this emission band is observed like a bandtail for TiCl_2_Pc. All of this may be related to the ligand coordinated to the tetravalent titanium affecting the number of intrinsic levels on the conduction bands. The emission doublet around 800–950 nm is also related to the phthalocyanine and is affected by the ligand by means of the exciton recombination. The broadening of both intense signals may be related to the formation of delocalized states between HOMO and LUMO, indicative of a non-radiative mechanism. For the PEDOT:PSS/TiCl_2_Pc (Figure 8b) and PEDOT:PSS/TiOPc films (Figure 8d), a change in the spectra is observed, compared to Figure 8a,c. A blue shift in the maximum emission band to 420 nm is observed. However, a shift to 410 nm is observed in Figure 8b for the near edge and edge locations, influenced more by the PEDOT:PSS due to the film deposition process. The emission bands around 420–450 nm and 760–810 nm are attributed to the PEDOT:PSS [54,55]. The PEDOT:PSS/TiCl_2_Pc spectra (Figure 7b) present a second emission band around 550 nm that can be related to the TiCl_2_Pc, which is more intense in the center location. Although the Q and B bands of phthalocyanine are primarily responsible for PL in films, according to our data, the PL in the films is substantially affected by the intermolecular structure. The smallest optical gap is obtained in the films with PEDOT:PSS and, in this case, the PL is enhanced by the presence of the polymer (Figure 8b,d).

### 3.3. Determination of Electrical Behavior

**Figure 9:**
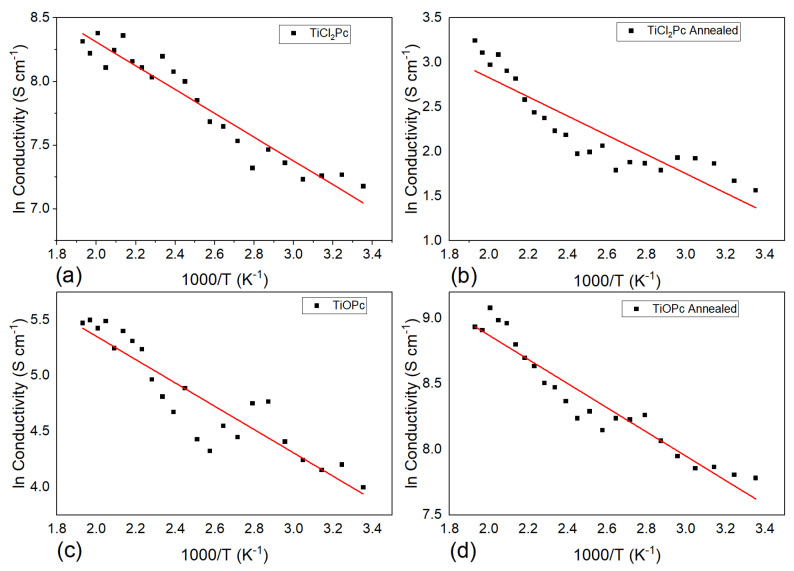
Arrhenius plot of TiCl_2_Pc and TiOPc before (**a**,**c**) and after annealing (**b**,**d**), respectively.

One-layer and two-layer planar heterojunction devices were constructed (see Figure 2) and electrical conductivities of the TiCl_2_Pc and TiOPc films with thermal annealing were obtained from I-V characteristics and measured in a 300–520 K temperature range (Figure 9). The obtained conductivities were around 10–10^3^ S/cm, presenting similar increasing behavior with temperature. It is interesting to note that the TiCl_2_Pc (Figure 9a) presents higher conductivities than TiOPc. The conductivity presents the following equation [56,57]:

(8) σ=σ0e−EakT

where *E_a_* is the thermal activation energy of the electrical conductivity, σ_0_ is the pre-exponential factor depending on the material nature, and k is Boltzmann’s constant (1.38 × 10^−23^ J/K). As observed in Figure 9, a plot of ln(*σ*) versus 1000/T was linearly fitted and the slope can be used to determine the thermal activation energies of the thin films. The conductivities are close to reported values for various Pcs [56,57]. The calculated activation energy values yield between 0.18 and 0.21 eV before and after thermal annealing, similar to reported PC results [53,54]. TiCl_2_Pc (0.185 eV) presents a lower activation energy than TiOPc (0.208 eV) (Figure 9a,c). However, after thermal annealing, TiCl_2_Pc (0.214 eV) presents a higher activation energy than TiOPc (0.183 eV). The previous may be related to a change in the molecular array and packing and to the film homogeneity due to film growth process.

The model fitting of null-ellipsometry measurements provided the optical properties of the films, as shown in Table 5. The ellipsometric parameters Psi and Delta were given by the change in the light polarization state due to the sample reflection and are related to the magnitude of reflectivity and the phase, respectively. The following equation describes the ratio of sample reflectivity:

(9) $$\rho =\frac{R_{p}}{R_{s}}=\tan\left(\psi \right)e^{i\Delta }$$

where *R_p_* and *R_s_* are the Fresnel reflection coefficients for the p- and s-polarized light. The incident light electric fields are parallel (*p*) and perpendicular (*s*) to the plane of incidence. Psi represents the amplitude ratio and delta the phase difference in the light polarization caused by the surface reflection. However, the refractive index can be obtained from the following equation:

(10) $$\langle \epsilon \rangle ={\left(\langle n\rangle +i\langle k\rangle \right)}^{2}=\sin{\left(\theta \right)}^{2}\left[1+\tan{\left(\theta \right)}^{2}{\left(\frac{1-\tan\left(\psi \right)e^{i\Delta }}{1+\tan\left(\psi \right)e^{i\Delta }}\right)}^{2}\right]$$

where *ε* is the dielectric function, *n* the refractive index, *k* the extinction coefficient, and *θ* the light incident angle. It can be observed that for Psi, the TiCl_2_Pc presents a larger value. However, the Psi magnitude is lower for the devices and the difference is larger among the devices, but the Psi magnitude is larger for the PEDOT:PSS-TiOPc device. Despite this, the delta parameter presents the opposite behavior, but also, the devices present smaller values. On the other hand, the films’ refractive index was also obtained from the previous parameters and is shown in Table 5. The small refractive index shown for the films is indicative of a small light reflection by passing light from an air medium (n = 1) to the films, allowing for a higher light absorption for solar cell applications. By comparing the devices, it is interesting to note that the refractive index is larger for the TiCl_2_Pc than for TiOPc and for both PEDOT:PSS devices, it is increased, but more pronounced for the PEDOT:PSS/TiCl_2_Pc. The obtained results are a good indicator that the devices with these active films should be used for photovoltaic applications.

**Figure 10:**
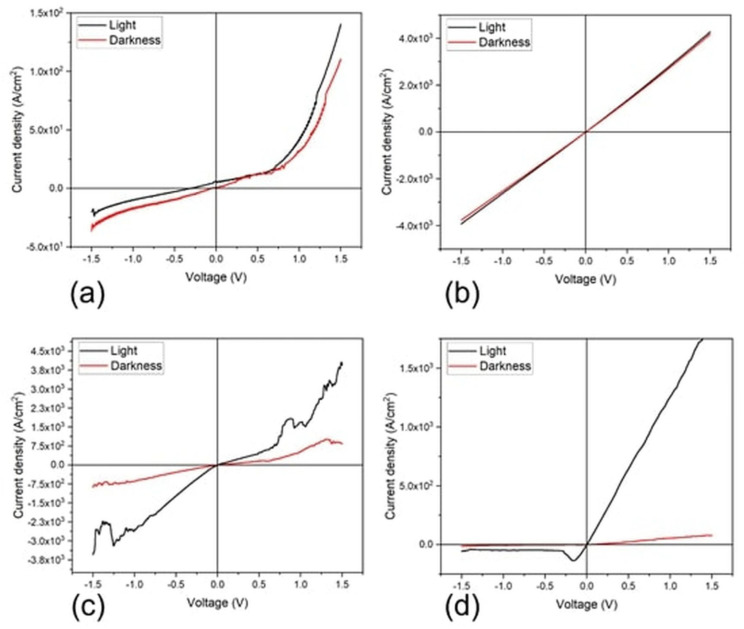
Darkness and illuminated J–V characteristic curve plot of (**a**) TiCl_2_Pc and (**b**) TiOPc and (**c**,**d**) with PEDOT:PSS devices, respectively.

The current density–voltage (J–V) measurements were initially performed at room temperature and in darkness, while also under illumination conditions for the device structures shown in Figure 2. The active film thickness was 5.8 nm and 22.7 nm for the MPc and PEDOT:PSS/MPc device type, respectively. The purpose was to compare the light effect on the film’s electrical behavior. Figure 10 presents the J–V characteristic curves obtained for the films and devices. First, the curves present different electrical behavior and are not symmetrical. A change in the J–V curves is observed for the illuminated condition compared to the darkness condition for all the devices; however, the effect depends on the device architecture. The latter indicates that the devices may be used for optoelectronic applications. The darkness and illuminated J–V characteristic curves for TiCl_2_Pc (Figure 10a) resemble a Schottky curve, which, under illuminated conditions, shows larger current density values, suitable for solar cell applications. The darkness curve shows, at 1.5 V, an approximate current density of 1.1 × 10^2^ A/cm^2^ compared to the 1.4×10^2^ A/cm^2^ of the light curve. However, the curves for TiOPc (Figure 10b) are very different to the TiCl_2_Pc, resulting in an almost linear behavior and larger current density values. Hence, there is an important effect of the ligand on the electrical output. Despite this, there is also a change in the current density values due to illumination of approximately 1.5 × 10^2^ A/cm^2^ at 1.5 V. On the other hand, the devices with PEDOT:PSS shown in Figure 10c,d present a larger effect on the current density due to illumination compared to the previous devices. Further, an important change in the curve shape and current density values is observed due to the PEDOT:PSS layer. In the case of the PEDOT:PSS/TiCl_2_Pc (Figure 10c), there is an enhancement in the current density values but, for the PEDOT:PSS/TiOPc, the opposite effect is observed. Table 5 presents the photocurrent density at 0 V for the different devices. It is observed that the device with PEDOT:PSS/TiOPc presents enhanced photocurrent compared to TiOPc, but the largest value is for the TiCl_2_Pc (6.56 A/cm^2^), while the smallest is for the TiOPc (0.03 A/cm^2^). A change in photocurrent of about 28-times between the device with and without PEDOT:PSS is observed for the TiOPc and of about 5-times for the TiCl_2_Pc.

**Figure 11:**
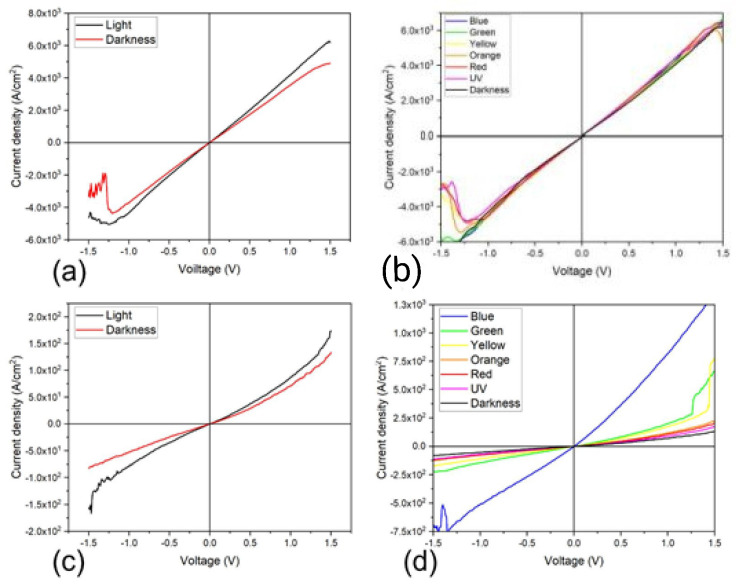
Darkness and illuminated J–V characteristic curves plot for annealed (**a**) PEDOT:PSS/TiCl_2_Pc and (**c**) PEDOT:PSS/TiOPc, and with different light colors (**b**,**d**), respectively.

**Figure 12:**
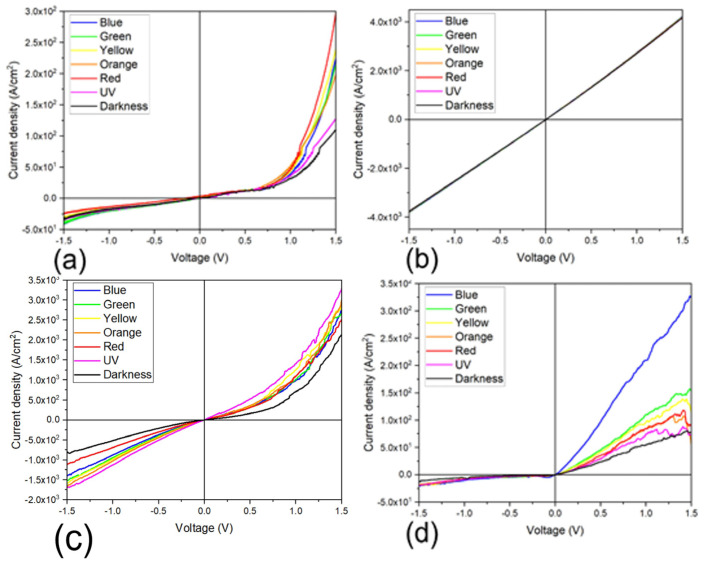
Different light color J–V characteristic curve plot of (**a**) TiCl_2_Pc and (**b**) TiOPc films and (**c**,**d**) devices, respectively.

Additionally, the PEDOT:PSS/TiCl_2_Pc and PEDOT:PSS/TiOPc devices were subjected to thermal annealing and J–V characteristic curves were obtained and plotted in Figure 11. Compared to devices with no annealing, the thermal annealing process increases the current density values, enhancing the conductivity and affecting the photocurrent derived from the illuminated conditions. The curves for darkness and illuminated conditions are not symmetrical and the current density values are larger for the light curves, also indicating that the annealed devices are suitable for optoelectronic applications. For further analysis, the devices were illuminated with different light colors and J–V curves were measured. Figure 11b,d and Figure 12 show the resulting characteristic curves for the different devices, including the darkness curve as a reference. Figure 12a shows the J–V curve for the TiCl_2_Pc and a marked effect due to the incident light color is observed, where the largest current density value variation is observed for the red light and the least for UV light. Figure 12b shows the J–V curve for the TiOPc and a slight effect due to the incident light color is observed. Figure 12c shows the J–V curve for the PEDOT:PSS/TiCl_2_Pc and also a marked effect due to the incident light color is observed. However, the largest effect is observed under UV and yellow incident lights. Figure 12d shows the J–V curve for the PEDOT:PSS/TiOPc, with a marked effect due to the incident light color, where the largest photocurrent density is observed for the blue light, while for the UV light, the lowest. However, an apparent increase in the photocurrent with the wavelength is observed, disregarding the UV curve. It is interesting to note that the blue-color curve presents a more pronounced photocurrent, which may indicate that the PEDOT:PSS/TiOPc is more photo-sensitive to this wavelength. For the PEDOT:PSS/TiCl_2_Pc annealed device (Figure 11b), the incident light effect in the photogenerated current is small but still observable and dependent on the light color. Further, for the PEDOT:PSS/TiOPc annealed device (Figure 10b), an incident light effect is observed, where the largest current density value variation is observed for the blue light and the least for the UV light. An almost direct relation to the wavelength variation is observed by not considering the UV curve.

**Figure 13:**
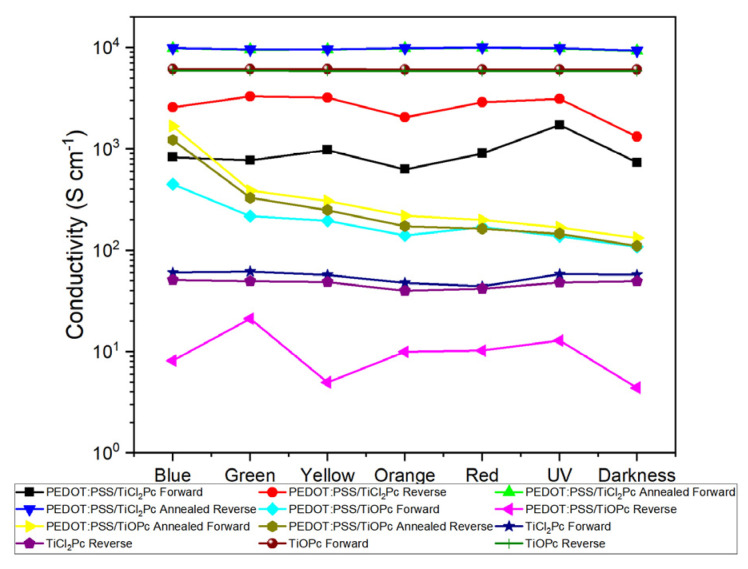
Forward and reverse bias device conductivity.

Moreover, the conductivity values on forward and reverse bias were calculated for the devices under different incident light colors, from the J–V characteristic curves, plotted in Figure 13. The obtained conductivity values lay between approximately 10 and 10^4^ S/cm, in good accordance with other Pc results in the literature [57,58]. It can be observed that depending on the device architecture and annealing, there is a change in the conductivity for forward and reverse bias. This variation may be significant, as for the PEDOT:PSS/TiOPc, and less so for the TiOPc. The greatest forward bias conductivity is observed for the annealed PEDOT:PSS/TiCl_2_Pc and the smallest for the TiCl_2_Pc, while for the reverse bias conductivity, the greatest is observed for the annealed PEDOT:PSS-TiCl_2_Pc and the smallest for the PEDOT:PSS/TiOPc. The effect of the incident light on the conductivity shown in Figure 13 indicates that some of the devices have no variation, whereas other devices are more affected and, in some cases, present a tendency, as for annealed PEDOT:PSS/TiOPc.

## 4. Conclusions

A comparative study between thin films of non-planar Titanyl phthalocyanine (TiOPc) and titanium(IV) phthalocyanine dichloride (TiCl_2_Pc) was carried out. The films were studied for their structure and morphology, as well as for their optoelectronic properties. These properties were enhanced by planar heterojunction formation with the PEDOT:PSS polymer. Bandgap, photoluminescence, activation energy, and current density values place these films as good active layers for photovoltaic devices.

## Figures and Tables

**Table 1:** Comparison between the IR signals obtained for TiOPc and TiCl_2_Pc in KBr pellet and film over silicon.

Assignment	TiOPc KBr Pellet (cm^−1^)	TiOPc As-Deposited Films (cm^−1^)	TiCl_2_Pc KBr Pellet (cm^−1^)	TiCl_2_Pc As-Deposited Films (cm^−1^)
C=C stretching	1611	1610	1611	1609
C=C benzene stretching	1476	1471	1472	1479
In-plane pyrrole stretching	1584, 1338	1591, 1331	1585,1334	1591, 1335
C-H bending	1293, 1166, 1118	1284, 1162, 1117	1293, 1160, 1119	1286, 1169, 1119
In plane C-H deformation	751	754	753	754

**Table 2:** Roughness and mechanical properties of the TiOPc and TiCl_2_Pc films.

Film	RMS (nm)	Ra (nm)
TiOPc	18.33	13.32
TiCl_2_Pc	17.67	14.61

**Table 3:** XRD peak position, FWHM, and crystallite size for the TiOPc and TiCl_2_Pc films and devices.

TiCl_2_Pc			TiOPc Annealed			TiCl_2_Pc Annealed			PEDOT:PSS/TiOPc			PEDOT:PSS/TiCl_2_Pc		
2θ (Degree)	FWHM (Degree)	D (nm)	2θ (Degree)	FWHM (Degree)	D (nm)	2θ (Degree)	FWHM (Degree)	D (nm)	2θ (Degree)	FWHM (Degree)	D (nm)	2θ (Degree)	FWHM (Degree)	D (nm)
-	-	-	7.680	0.407	0.340	7.611	0.611	0.226	7.630	0.164	0.843	9.620	0.856	0.162
-	-	-	-	-	-	-	-	-	9.360	0.553	0.251	-	-	-
11.020	0.393	0.355	-	-	-	-	-	-	-	-	-	10.619	0.585	0.238
-	-	-	12.781	0.575	0.244	12.792	0.398	0.353	13.582	0.124	1.137	-	-	-
17.977	0.324	0.445	-	-	-	-	-	-	-	-	-	-	-	-
19.280	0.628	0.231	-	-	-	-	-	-	-	-	-	-	-	-
24.740	0.462	0.327	25.560	0.377	0.403	25.740	0.605	0.252	26.315	0.225	0.680	26.635	0.523	0.293
26.783	0.404	0.380	-	-	-	-	-	-	-	-	-	-	-	-
28.688	0.337	0.464	28.573	0.378	0.413	28.669	0.638	0.245	27.334	0.217	0.711	-	-	-
29.321	0.297	0.529	31.081	0.380	0.421	-	-	-	33.014	0.144	1.135	-	-	-
34.280	0.366	0.453	-	-	-	-	-	-	-	-	-	33.265	0.154	1.064
-	-	-	47.982	0.352	0.582	46.100	0.362	0.546				-	-	-
-	-	-	49.660	0.456	0.464	-	-	-	-	-	-	-	-	-
-	-	-	50.980	0.287	0.759	-	-	-	-	-	-	-	-	-
-	-	-	-	-	-	60.674	0.458	0.611	61.740	0.163	1.776	61.814	0.214	1.356
-	-	-	65.791	0.324	1.032	-	-	-	65.964	0.144	2.337	-	-	-
-	-	-	-	-	-	-	-	-	66.526	0.133	2.587	-	-	-

**Table 4:** Onset gap $\left(E_{g}^{onset}\right)$ and optical gap $\left(E_{g}^{optical}\right)$ for the TiOPc and TiCl_2_Pc films.

Thin Film	Onset Gap (eV)	Optical Gap (eV)
TiOPc	1.43	2.85
TiOPc heat treated	1.32	2.85
TiOPc + PEDOT:PSS	1.5	2.91
TiOPc + PEDOT:PSS heat treated	1.27	2.85
TiCl_2_Pc	1.48	2.83
TiCl_2_Pc heat treated	1.34	2.85
TiCl_2_Pc + PEDOT:PSS	1.52	2.94
TiCl_2_Pc + PEDOT:PSS heat treated	1.29	2.79

**Table 5:** Optical and electrical properties for the TiOPc and TiCl_2_Pc films and devices.

	TiOPc	TiCl_2_Pc	PEDOT:PSS/TiOPc	PEDOT:PSS/TiCl_2_Pc
Psi (°)	25.00	28.80	22.70	6.50
Delta (°)	140.40	137.00	134.80	160.00
Refractive Index (n)	1.137	1.182	1.148	1.436
Photocurrent density (@ 0V, A/cm^2^)	0.03	6.56	0.84	1.45

## Data Availability

Data are contained within the article.
